# Glycyrrhizin Attenuates Portal Hypertension and Collateral Shunting via Inhibition of Extrahepatic Angiogenesis in Cirrhotic Rats

**DOI:** 10.3390/ijms22147662

**Published:** 2021-07-17

**Authors:** Chon Kit Pun, Hui-Chun Huang, Ching-Chih Chang, Chiao-Lin Chuang, Chun-Hsien Yen, Shao-Jung Hsu, Fa-Yauh Lee, Ming-Chih Hou, Yi-Hsiang Huang

**Affiliations:** 1 Faculty of Medicine, National Yang-Ming University School of Medicine, Taipei 11217, Taiwan; ckpan2@vghtpe.gov.tw (C.K.P.); hchuang2@vghtpe.gov.tw (H.-C.H.); ccchang7@vghtpe.gov.tw (C.-C.C.); clchuang@vghtpe.gov.tw (C.-L.C.); fylee@vghtpe.gov.tw (F.-Y.L.); mchou@vghtpe.gov.tw (M.-C.H.); yhhuang@vghtpe.gov.tw (Y.-H.H.); 2 Faculty of Medicine, National Yang Ming Chiao Tung University, Taipei 11217, Taiwan; 3 Division of Gastroenterology and Hepatology, Department of Medicine, Taipei Veterans General Hospital, Taipei 11217, Taiwan; sam1022yen86@gmail.com; 4 Division of General Medicine, Department of Medicine, Taipei Veterans General Hospital, Taipei 11217, Taiwan

**Keywords:** liver cirrhosis, portal hypertension, angiogenesis, portosystemic collateral shunting, glycyrrhizin

## Abstract

Portal hypertension develops along with liver cirrhosis then induces the formation of portal-systemic collaterals and lethal complications. Extrahepatic angiogenesis plays an important role. Glycyrrhizin has been found to exhibit anti-angiogenic features, which leads to its extensive use. However, the relevant effects of glycyrrhizin on liver cirrhosis and portal hypertension have not been evaluated. This study thus aimed to investigate the impact of glycyrrhizin on portal hypertension-related derangements in cirrhotic rats. Male Sprague-Dawley rats received bile duct ligation (BDL) to induce cirrhosis or sham operation as control. The rats were subdivided to receive glycyrrhizin (150 mg/kg/day, oral gavage) or vehicle beginning on the 15th day post operation, when BDL-induced liver fibrosis developed. The effects of glycyrrhizin were determined on the 28th day, the typical timing of BDL-induced cirrhosis. Glycyrrhizin significantly reduced portal pressure (*p* = 0.004). The splanchnic inflow as measured by superior mesenteric arterial flow decreased by 22% (*p* = 0.029). The portal-systemic collateral shunting degree reduced by 30% (*p* = 0.024). The mesenteric angiogenesis and phospho-VEGFR2 protein expression were also downregulated (*p* = 0.038 and 0.031, respectively). Glycyrrhizin did not significantly influence the liver biochemistry data. Although glycyrrhizin tended to reverse liver fibrosis, statistical significance was not reached (*p* = 0.069). Consistently, hepatic inflow from portal side, hepatic vascular resistance, and liver fibrosis-related protein expressions were not affected. Glycyrrhizin treatment at the stage of hepatic fibrosis still effectively attenuated portal hypertension and portosystemic collateral shunting. These beneficial effects were attributed to, at least in part, the suppression of mesenteric angiogenesis by VEGF signaling pathway downregulation.

## 1. Introduction

Portal hypertension, a phenomenon attributed to both systematic and portal hemodynamic derangements, develops along with the progression of liver cirrhosis. These hemodynamic changes include increased splanchnic blood flow, portal inflow, and enhanced hepatic vascular resistance, which lead to excess blood retention in the portal system [1]. To drain the stagnant blood out from the portal system to systemic circulation, portal-systemic collateral vasculature gradually forms. Nevertheless, portal-systemic collateral in itself poses threats such as gastroesophageal variceal hemorrhage and hepatic encephalopathy. Traditionally, the excess portal inflow was mainly due to overt splanchnic vasodilatation. Recently, evidence suggested that angiogenesis, the generation of new blood vessels, participates in the formation of portal-systemic collaterals, aggravation of splanchnic hyperemia, and increase of portal inflow [2]. During the process, the actions of vascular endothelium growth factor (VEGF) and activation of its receptor, VEGF receptor 2 (VEGFR-2) are considered the major factors [3]. In brief, attenuation of angiogenesis is a reasonable strategy to ameliorate portal hypertension and its complications.

Glycyrrhizin, the major bioactive compound of licorice roots extraction, has been widely used as an herbal medicine for anti-tumor, anti-inflammation, and anti-virus therapy in Asia [4]. A clinical trial further demonstrated that licorice exposure increased large arterial stiffness and systemic vascular resistance [5]. Glycyrrhizin has also been proved to have a hepatic protection effect. Glycyrrhizin and its metabolite, glycyrrhetinic acid, ameliorated bile-induced hepatotoxicity in rats through inhibiting apoptosis and necrosis of hepatocytes [6]. In rat models with hepatic injury, glycyrrhizin reduced plasma levels of aspartate aminotransferase (AST) and alanine aminotransferase (ALT), indicators of liver injury, as compared with those of the vehicle control group [7,8]. Moreover, a randomized control trial showed that glycyrrhizin plus tenofovir significantly reduced serum AST and ALT levels and decreased MELD score compared with tenofovir alone in patients with chronic hepatitis B with severe acute exacerbation [9].

Interestingly, a recent study found that glycyrrhizin effectively suppresses angiogenesis. Glycyrrhizin inhibited tumor growth and angiogenesis in vivo, and attenuated migration, invasion, and tube formation of endothelial cells [10]. Another study showed that glycyrrhizin suppressed angiogenesis activity of endothelial cells. Furthermore, it inhibited tumor growth and neovascularization in mice [11]. Angiogenesis plays an important role in portal hypertension. Furthermore, glycyrrhizin also induced hepatic vascular relaxation in rats with CCL_4_-induced liver cirrhosis [12]. However, the effects of glycyrrhizin on chronic liver cirrhosis and portal hypertension-related derangements have not been surveyed.

We therefore hypothesized that glycyrrhizin may exert beneficial effects on portal hypertension. Via a rat model with liver cirrhosis and portal hypertension, glycyrrhizin was administered at the stage of liver fibrosis, which is more relevant to the clinical condition.

## 2. Results

### 2.1. Effects of Glycyrrhizin on Body Weight and Systemic Circulation

**Figure 1:**
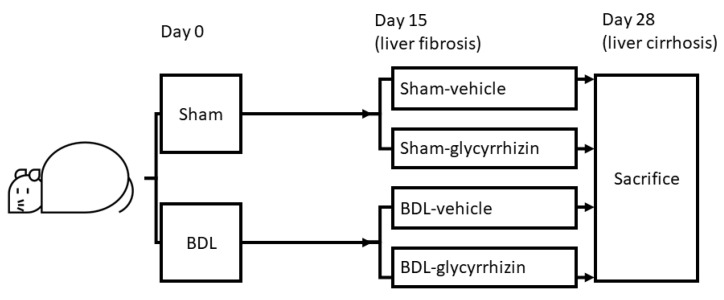
Experimental design. The rats received bile duct ligation (BDL) or sham operation. Treatments were started on the 15th day after operations, when liver fibrosis developed in BDL groups. After 2 weeks of treatments, experiments were performed on the 28th day after operations.

The rats received common bile duct ligation (BDL) to induce cirrhosis or sham operation as surgical control. The rats were then subdivided to receive glycyrrhizin or vehicle treatment beginning on the 15th day post operation, when BDL-induced liver fibrosis developed. Experiments were performed on the 28th day, when cirrhosis developed in the BDL group (Figure 1).

Table 1 depicts the results of BW and parameters of splanchnic and systemic circulation of experimental groups. The cirrhotic rats had significantly lower BW compared with sham-operated rats (*p* < 0.001). The MAP and SVR decreased while the CI increased significantly in cirrhotic groups, reflecting the feature of hyperdynamic circulation in portal hypertension (MAP: *p* = 0.012; SVR: *p* < 0.001; CI: *p* < 0.001). Glycyrrhizin treatment did not affect the BW, MAP, HR, SVR, and CI in sham-operated groups and BDL groups (*p* > 0.05).

### 2.2. Effects of Glycyrrhizin on Portal Hypertension

Compared with sham-operated groups, cirrhotic rats had significantly higher PP (*p* = 0.004). Glycyrrhizin treatment from the stage of liver fibrosis still significantly reduced PP in cirrhotic rats. In sham-operated groups, glycyrrhizin did not affect PP (*p* = 0.356).

### 2.3. Effects of Glycyrrhizin on Extrahepatic Systems and Mesenteric Angiogenesis

**Figure 2:**
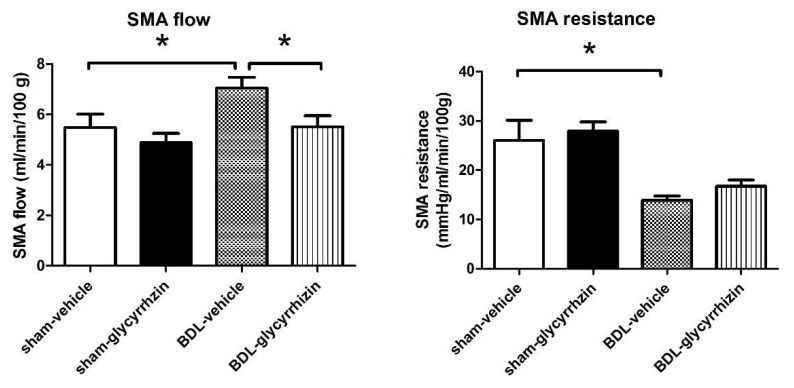
Effects of glycyrrhizin on splanchnic system. In rats with BDL-induced cirrhosis, the superior mesenteric artery (SMA) flow significantly increased compared with the sham-vehicle group. Glycyrrhizin significantly attenuated SMA flow in cirrhotic rats. The SMA resistance decreased in cirrhotic rats and was not affected by glycyrrhizin (*n* = 6, 6, 6, 6; BDL, bile duct ligation). * *p* < 0.05.

Portal hypertension is driven by abnormal splanchnic inflow and hepatic outflow. In cirrhotic (BDL)-vehicle rats, the SMA flow significantly increased compared with the sham-vehicle group, representing the abnormally high splanchnic blood flow (Figure 2, sham-vehicle vs. BDL-vehicle (mL/min/100 g): 5.48 ± 0.53 vs. 7.05 ± 0.42, *p* = 0.042). Consistently, the SMA resistance decreased in cirrhotic rats (sham-vehicle vs. BDL-vehicle (mmHg/mL/min/100 g): 26.0 ± 4.1 vs. 13.9 ± 0.8, *p* = 0.032). Glycyrrhizin significantly reduced SMA flow in cirrhotic rats (BDL-vehicle vs. BDL-glycyrrhizin: (mL/min/100 g): 7.05 ± 0.42 vs. 5.51 ± 0.44, *p* = 0.029). Interestingly, the SMA resistance was not affected by glycyrrhizin ((mmHg/mL/min/100 g): 13.9 ± 0.8 vs. 16.7 ± 1.3, *p* = 0.100).

**Figure 3:**
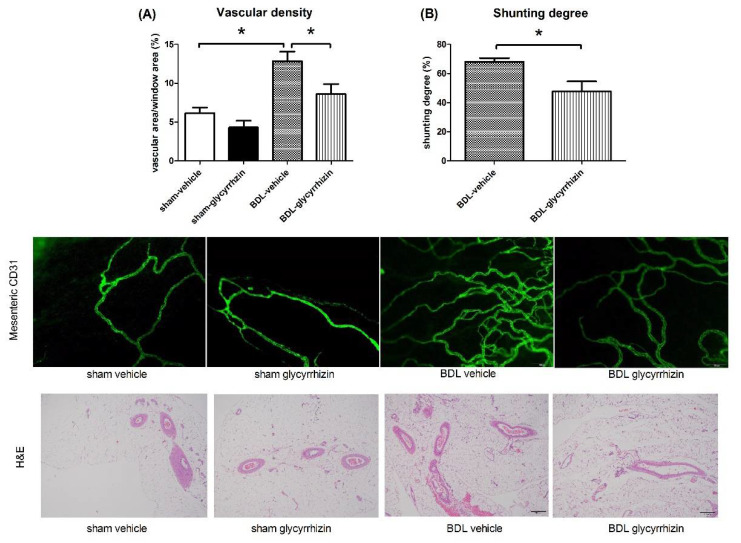
Effects of glycyrrhizin on mesenteric angiogenesis and portal-systemic collateral shunting. (**A**) Mesenteric angiogenesis was evaluated by vascular density of mesenteric window. In cirrhotic rats, the mesenteric vascular density was markedly higher than sham rats. Glycyrrhizin significantly reduced mesenteric vascular density (*n* = 5, 6, 6, 6). (**B**) The shunting degree was evaluated by color microsphere method. The shunting degree was significantly decreased in glycyrrhizin-treated group (*n* = 7, 7). Scale bar = 200 μm. * *p* < 0.05.

Mesenteric angiogenesis plays an important role in increasing splanchnic blood inflow and formation of portal-systemic collateral vascular system (Figure 3A). The mesenteric vascular density was evaluated by CD31 immunofluorescent staining (Figure 3B). In cirrhotic rats, the mesenteric vascular density was markedly higher than that of the sham-operated rats (sham-vehicle vs. BDL-vehicle (%): 6.2 ± 0.7 vs. 12.8 ± 1.2, *p* = 0.002). Glycyrrhizin treatment significantly reduced mesenteric vascular density (BDL-vehicle vs. BDL-glycyrrhizin (%): 12.8 ± 1.2 vs. 8.6 ± 1.3, *p* = 0.038). The collateral vascular system was evaluated by color microsphere method (Figure 3C). The results show that the shunting degree was significantly decreased in the glycyrrhizin-treated group ((%): 68.1 ± 2.5 vs. 47.8 ± 6.8, *p* = 0.024). This suggests that glycyrrhizin attenuates splanchnic blood inflow and portal-systemic collateral shunting though inhibition of splanchnic angiogenesis. This also explains why the SMA flow decreased in the glycyrrhizin-treated group with unaltered SMA resistance.

The angiogenic protein expressions of mesentery were assessed. A parallel series of experiments were performed to compare the protein expressions between sham-vehicle and BDL-vehicle groups (supplementary Figure S1). The results reveal that the phospho-eNOS, COX1, COX2, phospho-VEGFR2, and VEGF protein expressions significantly increased in the BDL group (sham-vehicle vs. BDL-vehicle, phospho-eNOS: 0.46 ± 0.15 vs. 1.00 ± 0.16, *p* = 0.032; COX1: 0.54 ± 0.10 vs. 0.80 ± 0.06, *p* = 0.047; COX2: 0.28 ± 0.02 vs. 0.58 ± 0.06, *p* = 0.001; phospho-VEGFR2: 0.49 ± 0.09 vs. 0.79 ± 0.04, *p* = 0.022; VEGF: 0.19 ± 0.03 vs. 0.78 ± 0.06, *p* < 0.001).

**Figure 4:**
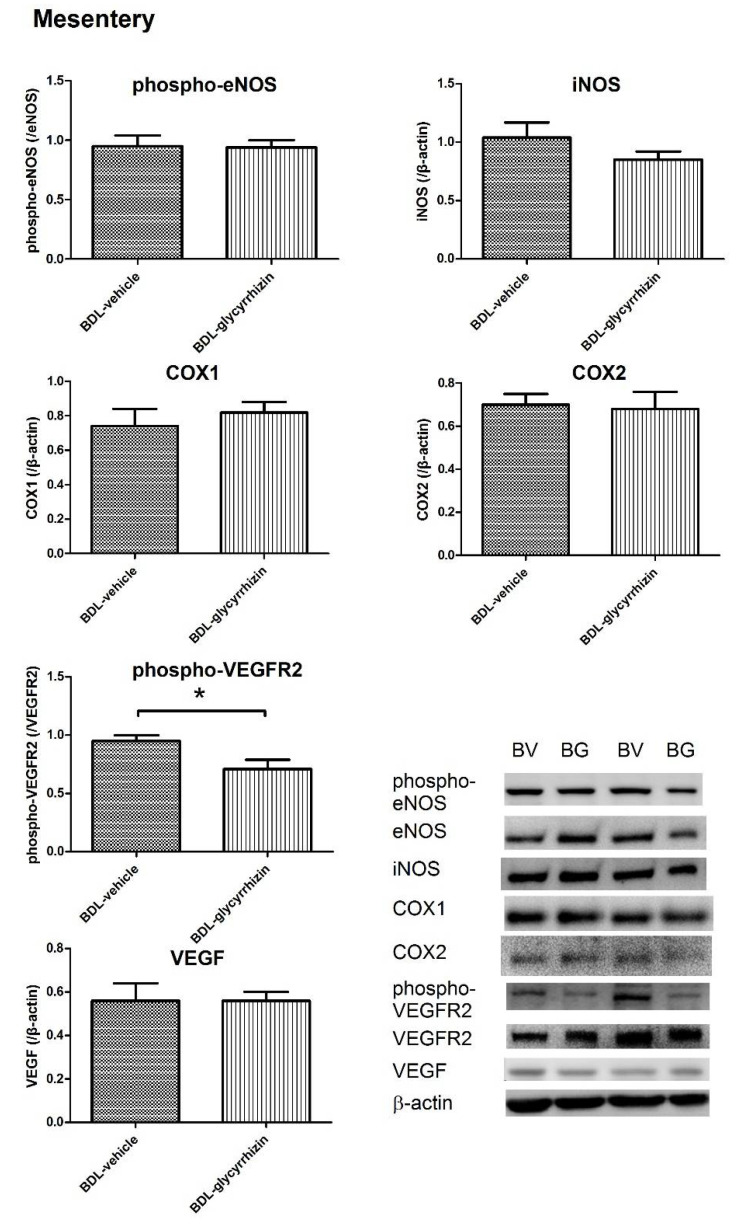
The angiogenic protein expressions in the mesentery. Glycyrrhizin downregulated phospho-VEGFR2 expression. VEGF, Phospho-eNOS, iNOS, COX1, and COX2 expressions were unaffected (*n* = 6, 6; BC, BDL-control; BG, BDL-glycyrrhizin). * *p* < 0.05.

Figure 4 discloses the protein expression of BDL groups that received vehicle or glycyrrhizin. The results show that phospho-VEGFR2 was significantly downregulated by glycyrrhizin ((/β-actin): BDL-vehicle vs. BDL-glycyrrhizin: 0.95 ± 0.05 vs. 0.71 ± 0.08, *p* = 0.031). On the other hand, VEGF, phospho-eNOS, iNOS, COX1, and COX2 expressions were unaffected (VEGF: 0.56 ± 0.08 vs. 0.56 ± 0.04, *p* = 0.996; phospho-eNOS: 1.00 ± 0.04 v.s. 1.00 ± 0.01, *p* = 0.954; iNOS: 1.04 ± 0.13 v.s. 0.85 ± 0.07, *p* = 0.211; COX1: 0.74 ± 0.10 vs. 0.82 ± 0.06, *p* = 0.534; COX2: 0.70 ± 0.05 v.s. 0.68 ± 0.08, *p* = 0.882). The uncropped membranes are shown in supplementary Figure S2.

### 2.4. Effects of Glycyrrhizin on Hepatic System

**Figure 5:**
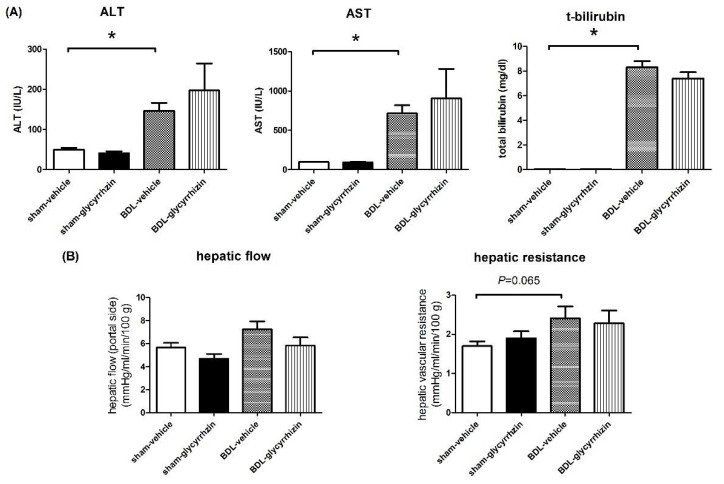
Effects of glycyrrhizin on hepatic system. (**A**) The levels of plasma liver injury markers alanine transaminase (ALT), aspartate transaminase (AST), and total bilirubin were determined. The levels of liver injury markers increased markedly in BDL groups. However, glycyrrhizin did not affect ALT, AST, and total bilirubin levels in cirrhotic rats (*n* = 6, 6, 6, 6). (**B**) There was a trend toward increased hepatic vascular resistance (HVR) in cirrhotic rats compared with sham-operated control rats. The HVR of the BDL-vehicle and BDL-glycyrrhizin groups was not significantly different (*n* = 6, 6, 6, 6). * *p* < 0.05.

The plasma liver injury markers ALT, AST, and total bilirubin were determined as well and shown in Figure 5A. The ALT, AST, and total bilirubin levels significantly increased in BDL groups (sham-vehicle vs. BDL-vehicle, ALT (IU/L): 50 ± 5 vs. 146 ± 20, *p* = 0.001; AST (IU/L): 97 ± 5 vs. 714 ± 105, *p* < 0.001; total bilirubin (mg/dl): 0.04 ± 0.01 vs. 8.30 ± 0.54, *p* < 0.001). However, glycyrrhizin did not influence ALT, AST, and total bilirubin levels in cirrhotic rats (BDL-vehicle vs. BDL-glycyrrhizin: ALT (IU/L): 146 ± 20 vs. 197 ± 67, *p* = 0.479; AST (IU/L): 714 ± 105 vs. 906 ± 372, *p* = 0.630; total bilirubin (mg/dl): 8.30 ± 0.54 vs. 7.37 ± 0.52, *p* = 0.244).

Figure 5B shows the hemodynamic parameters of the hepatic system. The HVR tended to increase in cirrhotic rats compared with sham-operated control rats (sham-vehicle vs. BDL-vehicle (mmHg/mL/min/100 g): 1.7 ± 0.1 vs. 2.4 ± 0.3, *p* = 0.065). There was no significant difference between the BDL-control and BDL-glycyrrhizin group in HVR ((mmHg/mL/min/100 g): 2.4 ± 0.3 vs. 2.3 ± 0.3, *p* = 0.785).

**Figure 6:**
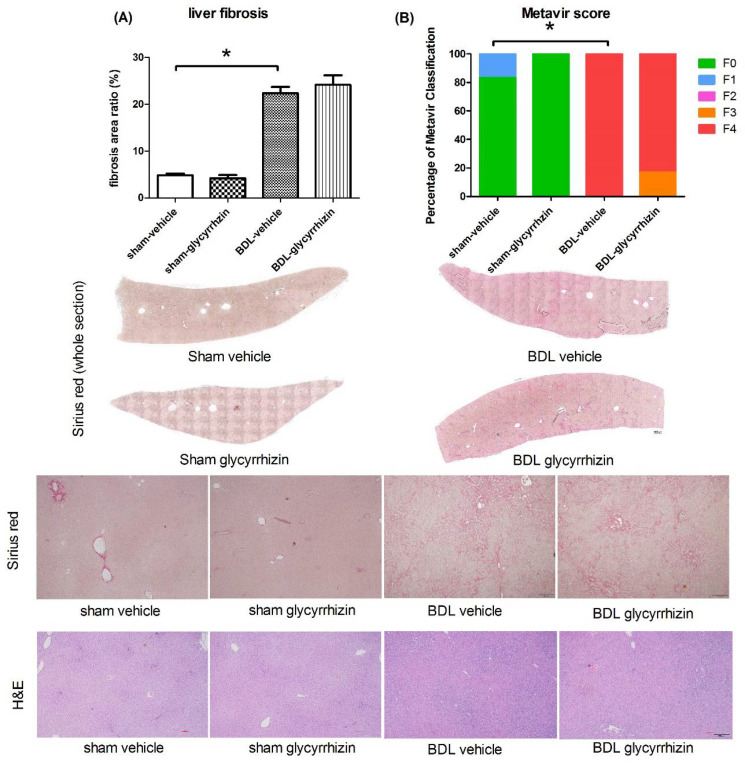
Effects of glycyrrhizin on liver fibrosis. (**A**) The severity of liver fibrosis was evaluated by fibrosis area ratio. The area ratio was significantly increased in BDL-vehicle group compared with sham-vehicle group. Glycyrrhizin treatment tended to attenuate the fibrosis severity (*n* = 6, 6, 6, 6). (**B**) The severity of liver fibrosis was further classified blindly by Metavir scoring system by an independent investigator. The results were compatible with the findings of the area ratio analyses that BDL significantly increased the fibrosis severity. Glycyrrhizin did not affect liver fibrosis in sham or BDL groups (*n* = 6, 6, 6, 6). Scale bar of upper panel = 1000 μm, scale bar of lower panel = 200 μm. * *p* < 0.05.

The severity of liver fibrosis was evaluated by fibrosis area ratio of the whole liver section, stained by Sirius red (Figure 6A). The area ratio was significantly increased in BDL-control group compared with the sham-control group ((%) 4.8 ± 0.4 vs. 22.4 ± 1.3, *p* < 0.001). Glycyrrhizin did not affect liver fibrosis (22.4 ± 1.3 vs. 24.2 ± 2.0, *p* = 0.476). To further validate the result, the severity of fibrosis of the liver sections were classified blindly by the Metavir scoring system by an independent investigator (Figure 6B). Consistently, BDL rats had significantly more severe liver fibrosis compared with sham rats (*p* = 0.002). Glycyrrhizin did not affect liver fibrosis in sham or BDL groups.

**Figure 7:**
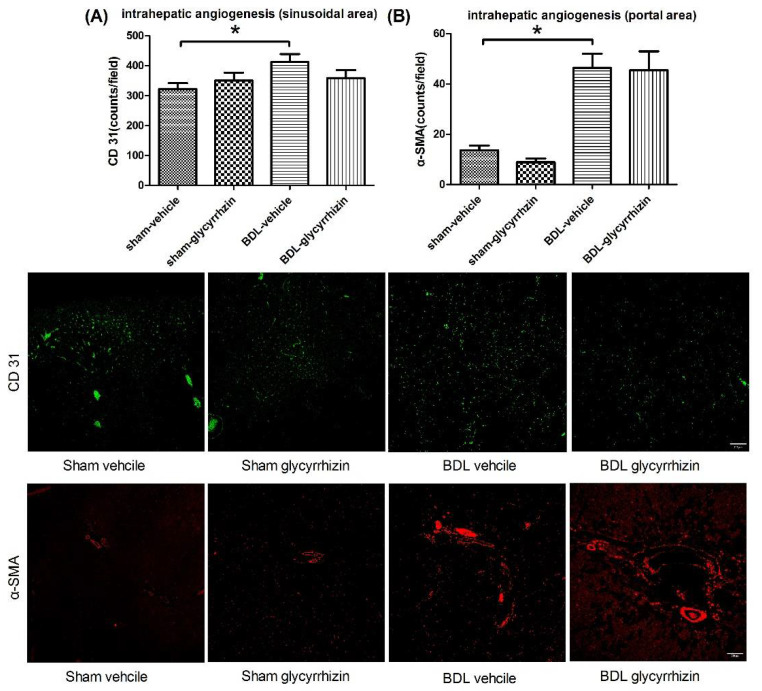
Effects of glycyrrhizin on intrahepatic angiogenesis. (**A**) Intrahepatic angiogenesis over sinusoidal area was evaluated by CD31 staining targeting endothelial cells. (**B**) Intrahepatic angiogenesis over portal area was investigated by α-SMA staining for vascular smooth muscle cells. The results were consistent. Intrahepatic vessels increased significantly in BDL-vehicle group compared with sham-vehicle group. Glycyrrhizin did not significantly influence the vascular numbers in the liver (*n* = 6, 6, 6, 6). Scale bar = 200 μm. * *p* < 0.05.

Intrahepatic angiogenesis was analyzed by comparing the vascular numbers in portal or sinusoidal area. In sinusoidal area, liver sinusoidal endothelial cells were stained with CD31 (Figure 7A). In the portal area, vessels were stained with α-SMA, a smooth muscle cell marker (Figure 7B). The two evaluation methods showed the consistent findings that there was significant intrahepatic angiogenesis in cirrhotic rats (sham-vehicle vs. BDL-vehicle, sinusoidal area (counts/field): 323 ± 19 vs. 420 ± 24, *p* = 0.010; portal area (counts/field): 14 ± 2 vs. 46 ± 6, *p* = 0.001). Glycyrrhizin did not affect intrahepatic angiogenesis (BDL-vehicle vs. BDL-glycyrrhizin, sinusoidal area (counts/field): 420 ± 24 vs. 360 ± 26, *p* = 0.121; portal area (counts/field): 46 ± 6 vs. 45 ± 8, *p* = 0.922). Intrahepatic angiogenesis promotes liver fibrosis and vice versa. This result further supports the non-significant effects of glycyrrhizin on intrahepatic circulation and liver fibrosis in this experimental setting.

The protein expressions of fibrogenesis factors in the liver of sham-vehicle and BDL-vehicle groups were investigated in another parallel series of experiments (supplementary Figure S3). In cirrhotic rats, α–SMA, procollagen Iα1, TIMP1, and TGF-β expressions increased and MMP13 decreased significantly (α–SMA: 0.21 ± 0.03 vs. 0.77 ± 0.10, *p* = 0.002; procollagen Iα1: 0.25 ± 0.03 vs. 0.57 ± 0.10, *p* = 0.020; MMP13: 0.72 ± 0.07 vs. 0.26 ± 0.06, *p* = 0.001; TIMP1: 0.34 ± 0.05 vs. 0.75 ± 0.10, *p* = 0.004; TGF-β: 0.34 ± 0.03 vs. 0.92 ± 0.05, *p* < 0.001).

**Figure 8:**
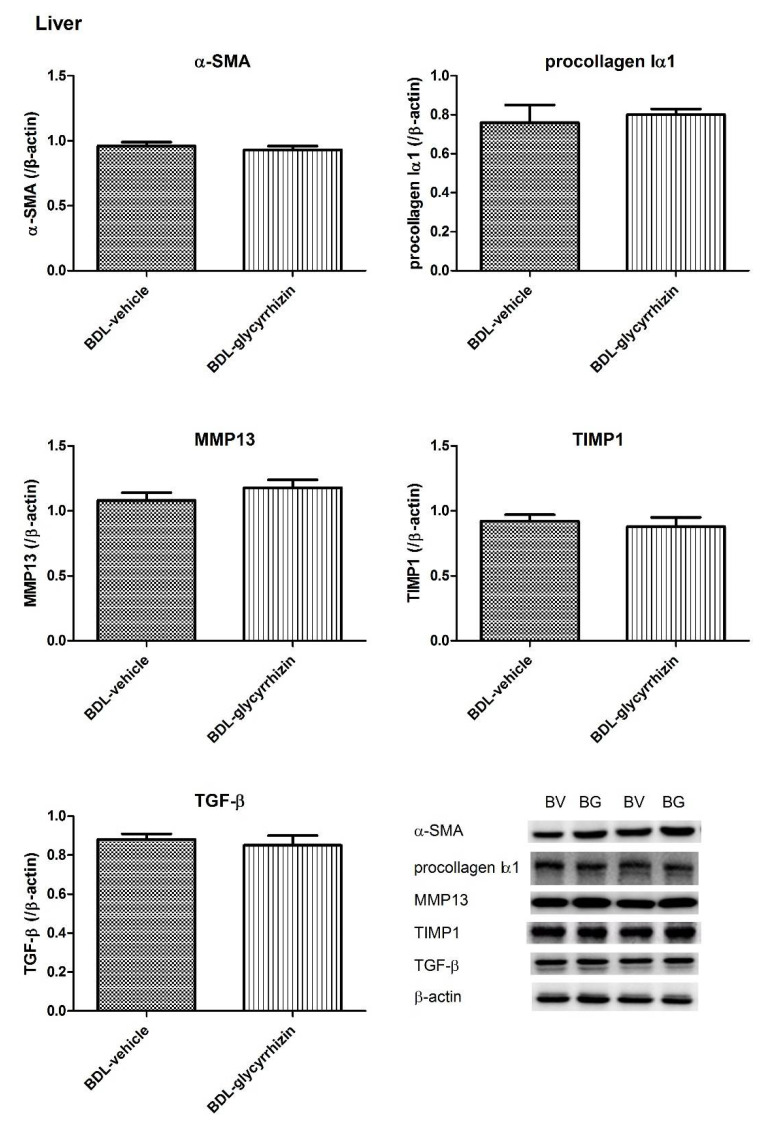
The fibrogenic protein expressions in the liver. The protein expressions of α–SMA, procollagen Iα1, MMP13, TIMP1, and TGF-β were not significantly different between BDL-vehicle (BV) and BDL-glycirrhizin (BG) groups (*n* = 6, 6).

The protein expressions were then determined in BDL-vehicle and BDL-glycyrrhizin groups (Figure 8). The protein expressions of α–SMA, procollagen Iα1, MMP13, TIMP1, and TGF-β in cirrhotic rats were not significantly influenced by glycyrrhizin (BDL-vehicle vs. BDL-glycyrrhizin (/β-actin): α–SMA: 0.96 ± 0.03 vs. 0.93 ± 0.03, *p* = 0.614; procollagen Iα1: 0.77 ± 0.09 vs. 0.80 ± 0.03, *p* = 0.747, MMP13: 1.08 ± 0.06 vs. 1.18 ± 0.06, *p* = 0.227; TIMP1: 0.92 ± 0.05 vs. 0.88 ± 0.07, *p* = 0.645; TGF-β: 0.88 ± 0.03 vs. 0.85 ± 0.05, *p* = 0.588). The results suggest that glycyrrhizin administration since the stage of liver fibrosis did not ameliorate the severity of fibrosis. The uncropped membranes are listed in supplementary Figure S4.

## 3. Discussion

**Figure 9:**
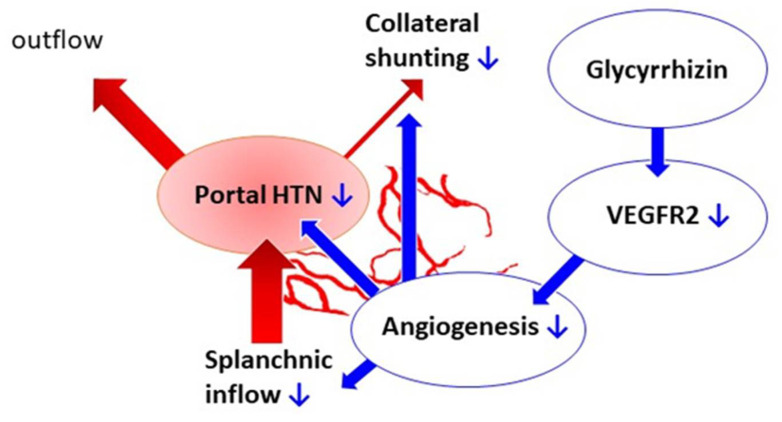
Effects of glycyrrhizin on portal hypertension-related derangements. Glycyrrhizin suppressed mesenteric angiogenesis through VEGFR2 downregulation. The splanchnic blood inflow and collateral shunting thus decreased. The hepatic system was unaffected by glycyrrhizin. The net effect of glycyrrhizin is the amelioration of portal hypertension.

In this study, glycyrrhizin effectively attenuated portal hypertension and portal-systemic collateral shunting degree. The actions and mechanism are shown in Figure 9. Glycyrrhizin was given starting at the 15th day after BDL, when liver fibrosis had developed. As a result, the findings could be more relevant to a clinical condition, implying that the administration of glycyrrhizin at the stage of liver fibrosis is still effective in ameliorating portal hypertension.

Portal hypertension develops along with the progression of liver cirrhosis. The collagen fiber in the liver interferes with the hepatic outflow. On the other hand, the splanchnic inflow increases pathologically. The abnormal blood flow becomes stagnant in the portal system and results in portal hypertension. To cope with the problem, the portal-systemic collateral vascular system develops to shunt the abnormal flow to the systemic circulation with a relatively “lower pressure”. Unfortunately, severe and fatal complications such as gastroesophageal variceal hemorrhage and hepatic encephalopathy ensue. Regarding the previous reports on the influences of glycyrrhizin in liver fibrosis, several studies have disclosed the anti-fibrotic effect of glycyrrhizin: Intraperitoneal injection of 3 mL of 0.2% glycyrrhizin solution three times a week beginning the first day of carbon tetrachloride administration markedly attenuated liver injury in a rat liver fibrosis model [13]. In another study, rats received carbon tetrachloride for 8 weeks to induce liver fibrosis. Treatment with glycyrrhizin beginning the first day of liver injury significantly ameliorated liver fibrosis [14]. Furthermore, in a mice liver fibrosis model induced by concanavalin A, glycyrrhizin treatment beginning the first day effectively ameliorated liver fibrosis [15]. In this study, glycyrrhizin treatment beginning at the stage of liver fibrosis did not significantly attenuate liver cirrhosis. Although the liver fibrosis ratio tended to decrease in the glycyrrhizin-treated group, further study showed that glycyrrhizin did not affect liver injury-related enzyme levels and liver fibrosis-related protein expressions. The contrary results may be ascribed to the timing to initiate treatment. Furthermore, the BDL-cirrhosis model adopted in this study leads to a relatively more severe liver damage as compared with that induced by carbon tetrachloride and concanavalin A [16]. Taken together, glycyrrhizin exerts a neutral effect on liver fibrosis under the current experimental setting. Indeed, there are several animal models of liver cirrhosis with various features. We chose the BDL model because it is a reproducible and nontoxic model. Since BDL-induced cholestatic liver injury may not be representative of all clinical conditions, other animal models simulating various liver injuries may be worth investigating in the future.

Abnormal angiogenic activity in splanchnic circulation further deteriorates portal hypertension. The portal blood flow is mainly supplied by the splanchnic system. During cirrhosis progression, abnormal angiogenesis in the splanchnic system further aggravates portal inflow [17]. Indeed, it has been demonstrated that inhibition of mesenteric angiogenesis ameliorated portal hypertension [3]. Several studies support that glycyrrhizin decreased angiogenesis activity. Glycyrrhizin also suppressed tumor growth and angiogenesis in mice [11]. In a rat colon precancerous model, glycyrrhizin suppressed the growth of lesions by the inhibition of angiogenesis [18]. In this study, glycyrrhizin decreased mesenteric vascular density, which suggests that glycyrrhizin effectively attenuated extrahepatic angiogenesis.

Activation of VEGFR2 is considered the main trigger of the most important pathway of angiogenesis in liver cirrhosis [3]. Fernandez et al. demonstrated that pharmaceutical blockade, either by antagonist or monoclonal antibody of the VEGF signaling molecules or receptors, effectively impeded neovascularization of portal-systemic collaterals and decreased portal blood inflow in rats with portal hypertension [17]. Our group has also identified that caffeine, through perturbing the VEGF signaling, exhibits beneficial effects toward hemodynamic derangements [19]. Taken together, the current and previous studies support the idea that extrahepatic angiogenesis due to VEGF pathway upregulation plays an important role in portal hypertension and manifests VEGF signaling antagonism as a potent therapeutic strategy in portal hypertension.

Several studies revealed the anti-VEGF properties of glycyrrhizin. Oral gavage of glycyrrhizin diminished VEGF in DMH-induced precancerous lesions in the colon of rats [18]. Glycyrrhizin significantly suppressed advanced glycation end product-induced VEGF production in rat retinal ganglion cell line [20]. Furthermore, glycyrrhizin attenuated VEGF and its receptor expression in a mice model with mammary cancer [21]. In this study, the protein expression of phospho-VEGFR2 in mesentery decreased significantly after glycyrrhizin administration. This finding supports prior research showing that glycyrrhizin inhibits angiogenesis via inhibition of the VEGF pathway.

The role of the spleen in portal hypertension should also be taken into consideration: Splenomegaly and increased splenic blood flow are important features of cirrhotic patients with portal hypertension. Increased splenic blood flow contributes to increased portal venous blood inflow, splanchnic hyperemia and angiogenesis, and aggravation of portal-systemic collateral vasculature [22]. A link of splenic blood flow in the pathogenesis of portal hypertension has also been suggested: The portal hypotensive effect of terlipressin, a drug used to control gastroesophageal variceal hemorrhage, is correlated with a decrease in splenic blood flow [23]. Therefore, glycyrrhizin may reduce the splenic size and/or splenic venous blood flow along with the reduction of portal pressure, which is something worth investigating.

The portosystemic collateral system diverts abnormal blood flow from the portal system. However, it results in severe complications such as esophageal variceal bleeding and hepatic encephalopathy. In this study, glycyrrhizin markedly attenuated collateral shunting. Since we have found that glycyrrhizin decreased splanchnic inflow but that the hepatic outflow (portal side) was not influenced, the current findings support the idea that the blood flow drained by the collateral system is reduced by glycyrrhizin, subsequently reducing the degree of collateral shunting.

## 4. Materials and Methods

### 4.1. Animal Model: Common Bile Duct Ligation (BDL)

Male Sprague-Dawley rats weighing 240–270 g at the time of surgery were used for experiments. The rats were allowed free access to food and water. Rats with secondary biliary cirrhosis were induced with common bile duct ligation [24]. Under anesthesia (Zoletil 50 mg/kg BW, intramuscularly), the common bile duct was doubly ligated with 3-0 silk. The first ligature was made below the junction of the hepatic ducts and the second ligature above the entrance of the pancreatic duct, followed by section of the common bile duct between the ligatures. The rats were allowed to recover. Liver cirrhosis developed two weeks after BDL, and a high yield of secondary biliary cirrhosis could be observed four weeks after BDL [25]. To avoid the coagulation defects, BDL rats received weekly vitamin K injection (50 μg/kg intramuscularly) [26].

This study was approved by Taipei Veterans General Hospital Animal Committee (IACUC 2017-081). All experimental procedures were performed at Taipei Veterans General Hospital Animal Laboratory and were conducted in accordance with the standard procedures indicated in the principles of laboratory animal care (Guide for the Care and Use of Laboratory Animals, DHEW publication No. (NIH) 85-23, rev. 985, Office of Science and Health Reports, DRR/NIH, Bethesda, MD, USA).

### 4.2. Experiment Design

Liver cirrhosis and portal hypertension were induced in male Sprague-Dawley rats through BDL. Sham-operated rats were controls. Sham and BDL rats receive glycyrrhizin (150 mg/kg/day, oral gavage) [27] or vehicle (distilled water) beginning at the 15th day after operations, when BDL-induced liver fibrosis had developed [26]. The effects of glycyrrhizin were evaluated on the 28th day.

### 4.3. Measurement of Systemic and Portal Hemodynamics

The right carotid artery was cannulated with a PE-50 catheter that was connected to a pressure transducer. Continuous recordings of mean arterial pressure (MAP), heart rate (HR), and PP were performed on a multi-channel recorder (MP45, Biopac Systems Inc., Goleta, CA, USA). The external zero reference was placed at the level of the mid-portion of the rat. The abdomen was then opened with a mid-line incision, and the mesenteric vein was cannulated with a PE-50 catheter connected to the transducer [28].

Superior mesenteric artery (SMA) was identified at its aortic origin and a 5 mm segment was gently dissected free from surrounding tissues. Then a pulsed-Doppler flow transducer (TS420, Transonic system Inc., Ithaca, NY, USA) was placed to measure the SMA flow [29]. Hepatic inflow via the portal vein (portal part) was also measured by placing a flow probe around the portal vein as proximal to the liver as possible.

Cardiac output (CO) was measured by thermodilution, as previously described [30]. Briefly, a thermistor was placed in the aortic arch just distal to the aortic valve, and the thermal indicator (100 μL of normal saline) was injected into the right atrium through a PE-50 catheter. The aortic thermistor was connected to a cardiac output computer Cardiomax III (Columbus Instruments International Co., Columbus, OH, USA). Five thermodilution curves were obtained for each cardiac output measurement. The final value was obtained from the arithmetic mean of the data. Cardiac index (CI, mL/min/100 g BW) was calculated as CO per 100 g BW. Systemic vascular resistance (SVR, mmHg/mL/min/100 g BW) was calculated by dividing MAP by CI. SMA resistance (mmHg/mL/min/100 g BW) was calculated by (MAP-PP)/SMA flow per 100 g BW. Hepatic vascular resistance (HVR, mmHg/mL/min/100 g BW) was calculated by PP/hepatic inflow (portal part) per 100 g BW.

### 4.4. Immunofluorescent Study for the Mesenteric Vascular Density

Mesenteric angiogenesis was quantified by CD31-labelled microvascular networks in rat mesenteric connective tissue windows according to the previous study [29]. From each rat, at least four mesenteric windows (wedge-shaped regions of connective tissue surrounded by the intestinal wall and the ileal blood vessel pairs) were dissected free, washed in PBS, dried on gelatin slides, and fixed in 100% MeOH (−20 °C for 30 min). Slides were then incubated overnight at 4 °C with the primary antibody mouse anti-rat CD31-biotin (AbD Serotec, Oxford, UK). Then, a secondary antibody (CY2-conjugated streptavidin; Jackson ImmunoResearch, West Grove, PA, USA) was applied for 1 h at room temperature. At least four sets of data were obtained for each mesenteric window. Immunofluorescent images at magnification ×100 were assessed using an upright fluorescent microscope (AX80, Olympus, Tokyo, Japan) and thresholded by ImageJ software (ImageJ, Available online: https://imagej.nih.gov/ij/ (accessed on 6 June 2018). The vascular area was measured with the histogram function.

### 4.5. Color Microsphere Method for Portosystemic Shunting Degree Analysis

Portosystemic shunting degree was determined using the technique described by Chojkier and Groszmann [31], substituting color for radioactive microspheres; 30,000 of 15 μm yellow microspheres (Dye Track; Triton Technology, San Diego, CA, USA) were slowly injected into the spleen. The rats were euthanized, and the livers and lungs were dissected and placed into new polypropylene centrifuge tubes. The number of microspheres in each tissue was determined following the protocol provided by the manufacturer. In brief, 3000 blue microspheres (Dye Track) were added to each tube as an internal control. Tissue was digested overnight with 1 M KOH at 60 °C and thoroughly sonicated. After centrifugation, the supernatant was removed, and the pellet was washed once with 10% Triton X-100 and twice with acidified ethanol. At the end of the process, a minimum pellet containing the microspheres was allowed to dry overnight. The color of the microspheres was diluted with 200 μL of acidified Cellosolve acetate (Spectrum Chemicals, Gardens, CA, USA). The absorbance of the solution was read at 448 nm wavelength (yellow) and 670 nm wavelength (blue) in a spectrophotometer (Shimadzu, Columbia, MD, USA), and the number of microspheres was calculated by comparison with standards. Spillover between wavelengths was corrected with the matrix inversion technique. Portosystemic shunting was calculated as lung microspheres/(liver microspheres plus lung microspheres). Assuming a worst-case scenario in which two-thirds of the microspheres remain trapped in the spleen, this technique detects a minimum shunt of 3.5%. Studies using color microspheres have been shown to provide results similar to those using radioactive microspheres [32].

### 4.6. Western Blot

Tissue was immediately frozen in liquid nitrogen and stored at −80 °C until required. The protein extracts were made by pulverization in a grinder with liquid nitrogen, using a ratio of 1 mL of lysis buffer (phosphate-buffered solution containing 1% Nonidet P-40, 0.5% sodium deoxycholate, 0.1% sodium dodecyl sulfate (SDS), and 0.05% protease inhibitor cocktail solution (Roche Diagnostics GmbH, Penzberg, Germany)) for each 100 mg powdered sample. Protein concentration was determined for each sample by the Bradford method [33]. An aliquot of 20–40 µg protein from each sample that dissolved in sample buffer (63 mmol/L of Tris-HCL, pH 6.8, containing 2% SDS, 10% glycerol, 5% 2-mercaptoethanol, and 0.005% bomphenol blue) and 10 µg positive control was separated on denaturing SDS-10% polyacrylamide gels by electrophoresis (Mini-PROTEAN^®^ 3 Cell, Bio-Rad Laboratories, Hercules, CA, USA). Prestained proteins markers (SDS-PAGE Standards, Bio-Rad Laboratories, Hercules, CA, USA) were used for molecular weight determinations. Proteins were then transferred to a polyvinylidene difluoride membrane (Immum-BlotTM PVDF Membrane, Bio-Rad Laboratories, Hercules, CA, USA) by a semi-dry electroblotting system (Trans-Blot^®^ SD Semi-dry Electrophoretic Transfer Cell, Bio-Rad Laboratories, Hercules, CA, USA) for 1.5 h at 4 °C. To block non-specific binding, membranes were blocked for 30 min with 3% non-fat dry milk in TBS-T, pH 7.4 (25 mmol/L Tris base-137 mmol/L NaCl-2.7 mmol/L KCL-1% Tween 20). Blots were incubated with the primary antibody, diluted with 3% non-fat dry milk in TBS-T for 90 min at room temperature, and washed. Then the blots were incubated for 90 min with the secondary antibody and washed. The specific proteins were detected by enhanced chemiluminescence (Immobilon Western Chemiluminescent HRP Substrate, Merk Millipore Co., Billerica, MA, USA) and scanned with a computer-assisted video densitometer and digitalized system (BioSpectrum^®^ 600 Imaging System, Ultra-Violet Products Ltd., Upland, CA, USA). The blots were scanned, photographed, then the signal intensity (integral volume) of the appropriate bands was analyzed.

### 4.7. Hepatic Fibrosis Determination with Sirius Red Staining

Liver paraffin sections were stained with Sirius red staining kit (Polysciences Inc., Warrington, PA, USA). To avoid selection bias, whole liver sections were analyzed. ImageJ was used to measure the percentage of Sirius red-stained area. Briefly, a grayscale image was used, then the red-stained collagen was isolated using the thresholding function. After that, the thresholded area was measured and shown as the percentage of thresholded area per image [29].

### 4.8. Drugs

Glycyrrhizin was purchased from Merck (Merck KGaA, Darmstadt, Germany). All solutions were freshly prepared on the days of the experiment.

### 4.9. Statistical Analysis

All results were analyzed using SPSS version 21.0 software [34] and data are expressed as mean ± S.E.M. The Shapiro–Wilk normality test showed that almost all of the data were in the pattern of “normal distribution”. This study was composed of 2 variables in 4 groups (sham vs. BDL and vehicle vs. glycyrrhizin). Therefore, we used unpaired Student’s *t*-test to check the differences between the following group pairs: 1. sham-vehicle vs. BDL-vehicle; 2. sham-vehicle vs. sham-glycyrrhizin; 3. BDL-vehicle vs. BDL-glycyrrhizin. The results of liver fibrosis severity classified by the Metavir scoring system were analyzed by Fisher’s exact test. Results were considered statistically significant at a two-tailed *p*-value less than 0.05.

## 5. Conclusions

In conclusion, glycyrrhizin administered even at the stage of fibrosis still effectively attenuated portal hypertension and portal-systemic collateral shunting. The beneficial effects were exerted, at least partly, through suppression of extrahepatic angiogenesis via VEGF pathway downregulation. The application of glycyrrhizin in liver cirrhosis in clinical settings deserves further investigation.

## Figures and Tables

**Table 1:** Hemodynamic parameters in sham or BDL rats treated with vehicle or glycyrrhizin.

	Sham Vehicle	Sham Glycyrrhizin	BDL Vehicle	BDL Glycyrrhizin	*p* Value *
	*n* = 6	*n* = 6	*n* = 6	*n* = 6	
BW (g)	452 ± 9	428 ± 15	375 ± 10 ^†^	386 ± 17	0.605
MAP (mmHg)	141 ± 8	142 ± 7	114 ± 4 ^†^	102 ± 4	0.075
HR (beats/min)	337 ± 19	304 ± 24	322 ± 13	302 ± 18	0.386
PP (mmHg)	9.5 ± 0.8	8.7 ± 0.4	16.8 ± 1.7 ^†^	12.3 ± 1.0 *	0.004
Systemic circulation					
CI (mL/min/100 g)	31.5 ± 1.4	33.2 ± 2.4	43.2 ± 1.4 ^†^	40.8 ± 3.7	0.560
SVR (mmHg/mL/min/100 g)	4.5 ± 0.3	4.3 ± 0.3	2.6 ± 0.1 ^†^	2.6 ± 0.3	0.976

BDL: common bile duct ligation; BW: body weight; MAP: mean arterial pressure; HR: heart rate; PP: portal pressure; CI: cardiac index; SVR: systemic vascular resistance. * Glycyrrhizin-treated groups compared with paralleled vehicle groups; † *p* < 0.05, BDL-vehicle group compared with sham-vehicle group.

## Data Availability

The datasets used and analyzed during the current study are available from the corresponding author on reasonable request.

## Supplementary Materials

The following are available online at https://www.mdpi.com/article/10.3390/ijms22147662/s1.
