# Supplementary material for: Glycyrrhizin Attenuates Portal Hypertension and Collateral Shunting via Inhibition of Extrahepatic Angiogenesis in Cirrhotic Rats
Source: Int J Mol Sci. 2021 Jul 17;22(14):7662. doi: 10.3390/ijms22147662 (PMC8304322; doi:10.3390/ijms22147662)

Supplementary Figures

Supplementary figure S1

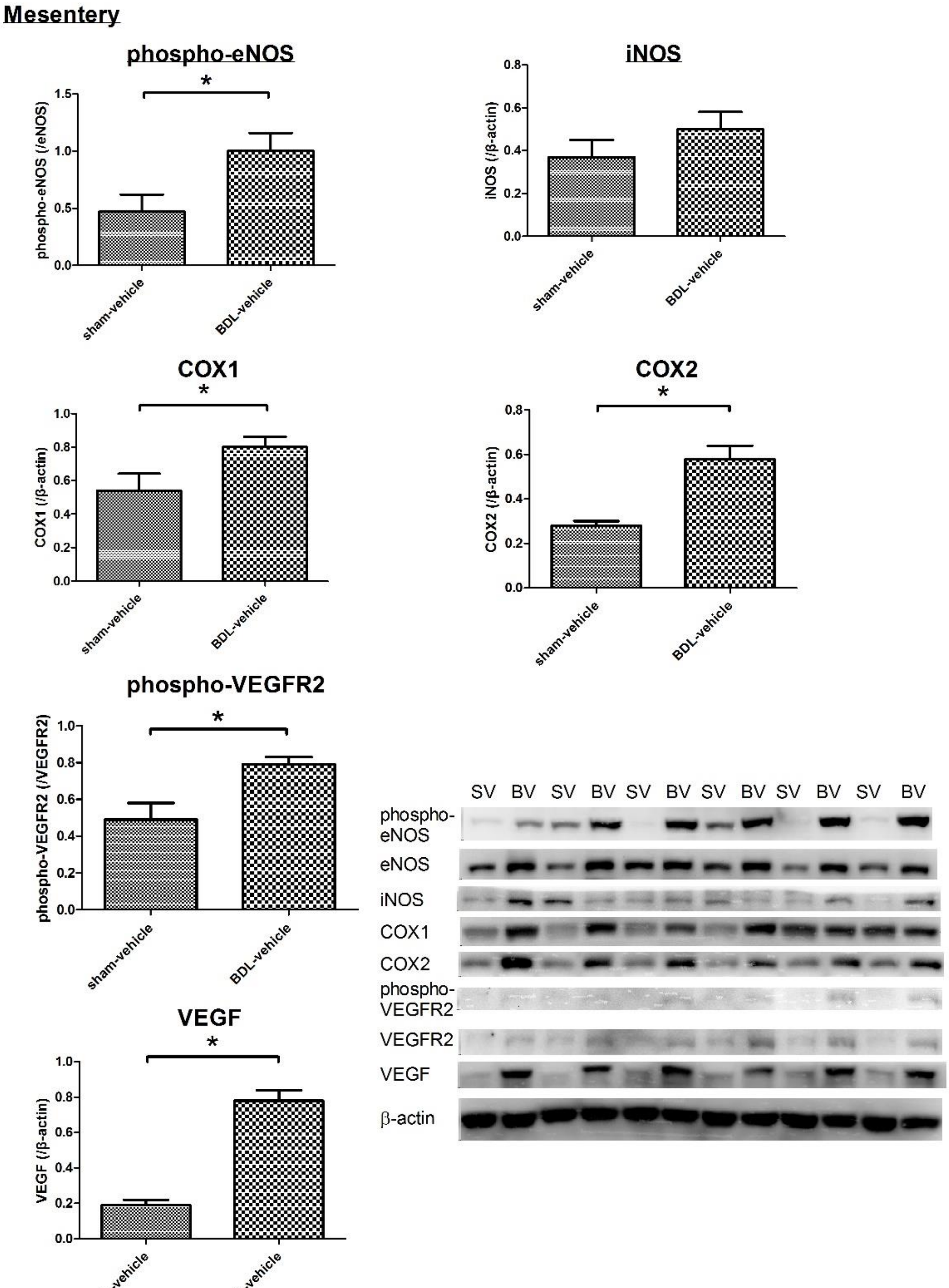

Supplementary figure S1. The angiogenic protein expressions in the mesentery. The protein expressions of phospho-eNOS, COX1, COX2, phospho-VEGFR2 and VEGF were significantly different between sham-vehicle (SV) and BDL-vehicle (BV) groups (n=6, 6). * P<0.05

Supplementary figure S2. Full uncropped Western blots of Mesentery (BDL-vehicle and BDL-glycyrrhizin groups)

Mesentery phospho-eNOS

Primary antibody: Genetex, GTX129058

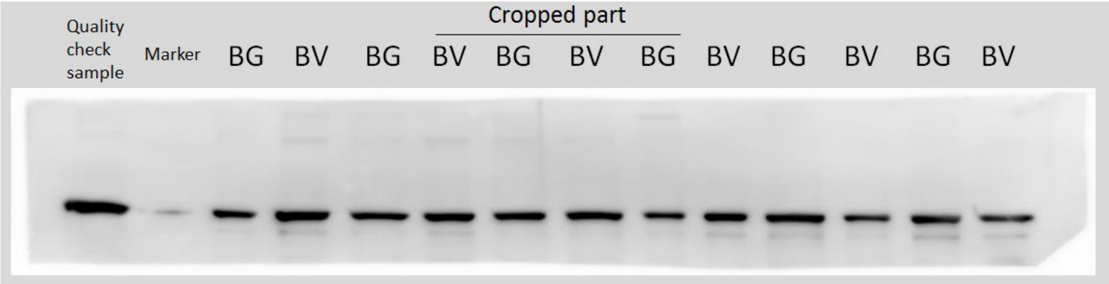

Mesentery eNOS

Primary antibody: Cell Signaling, #32027S

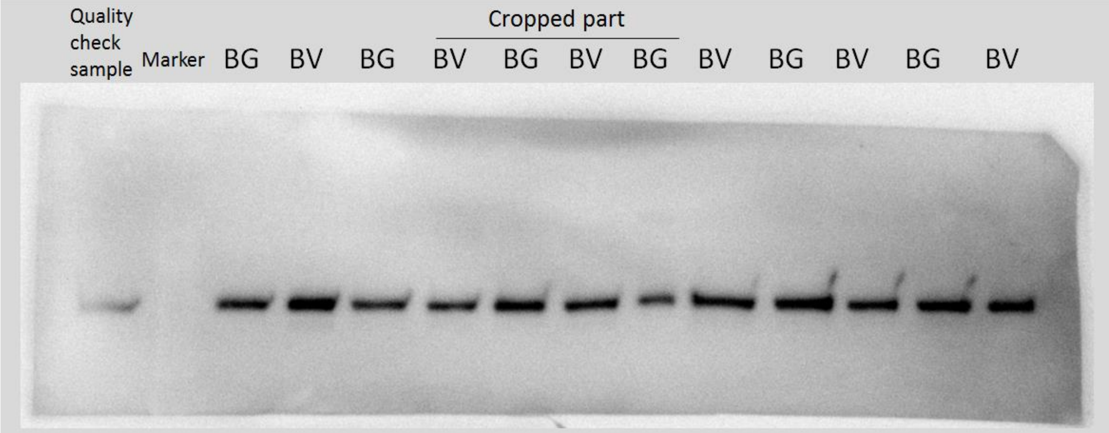

Mesentery iNOS

Primary antibody: Genetex, GTX130246

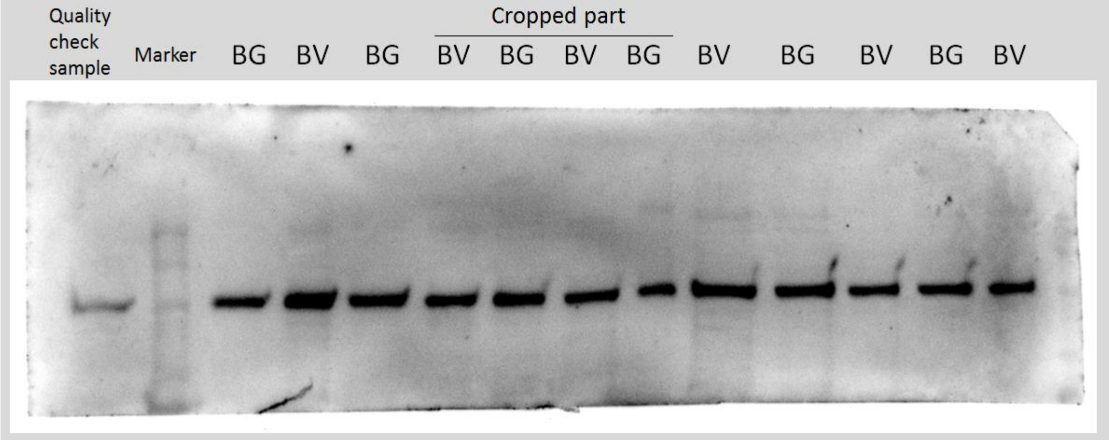

Mesentery COX1

Primary antibody: Cell Signaling, #4841S

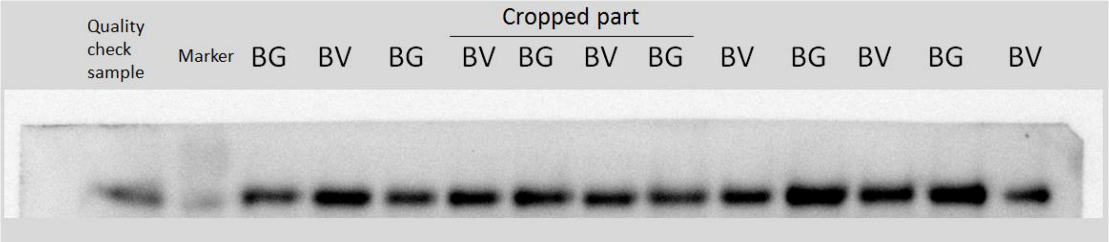

Mesentery COX2

Primary antibody: Cell Signaling, #12282S

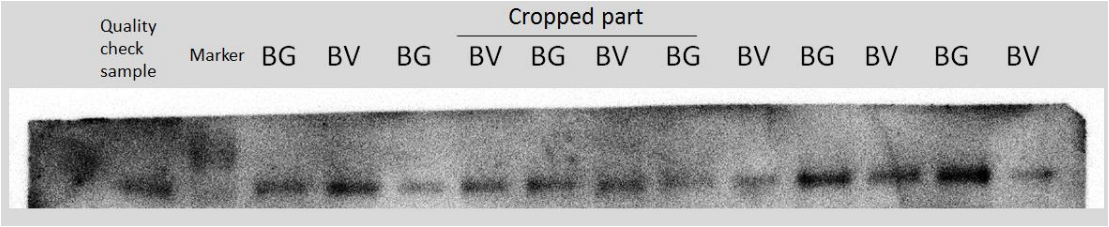

Mesentery Phospho-VEGFR2

Primary antibody: Genetex, GTX50153

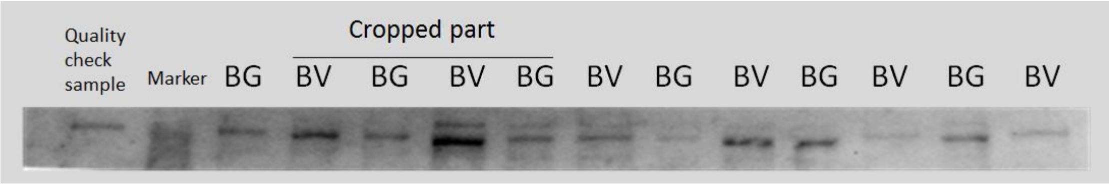

Mesentery VEGFR2

Primary antibody: Cell Signaling, #9698

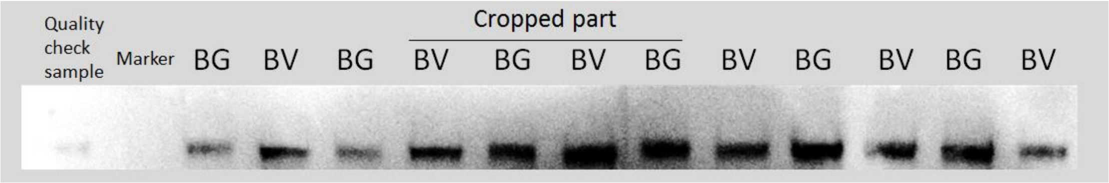

Mesentery β-actin

Primary antibody: Genetex, GTX629630

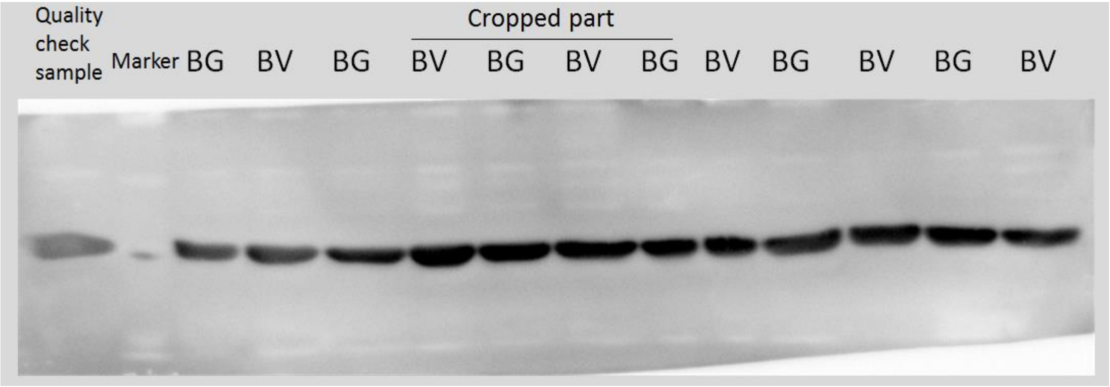

Mesentery VEGF

Primary antibody: Merch Millipore, ABS82

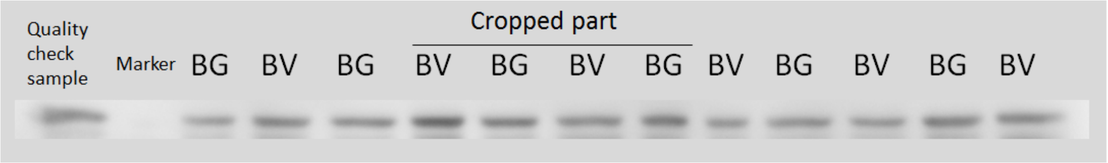

Supplementary figure S3

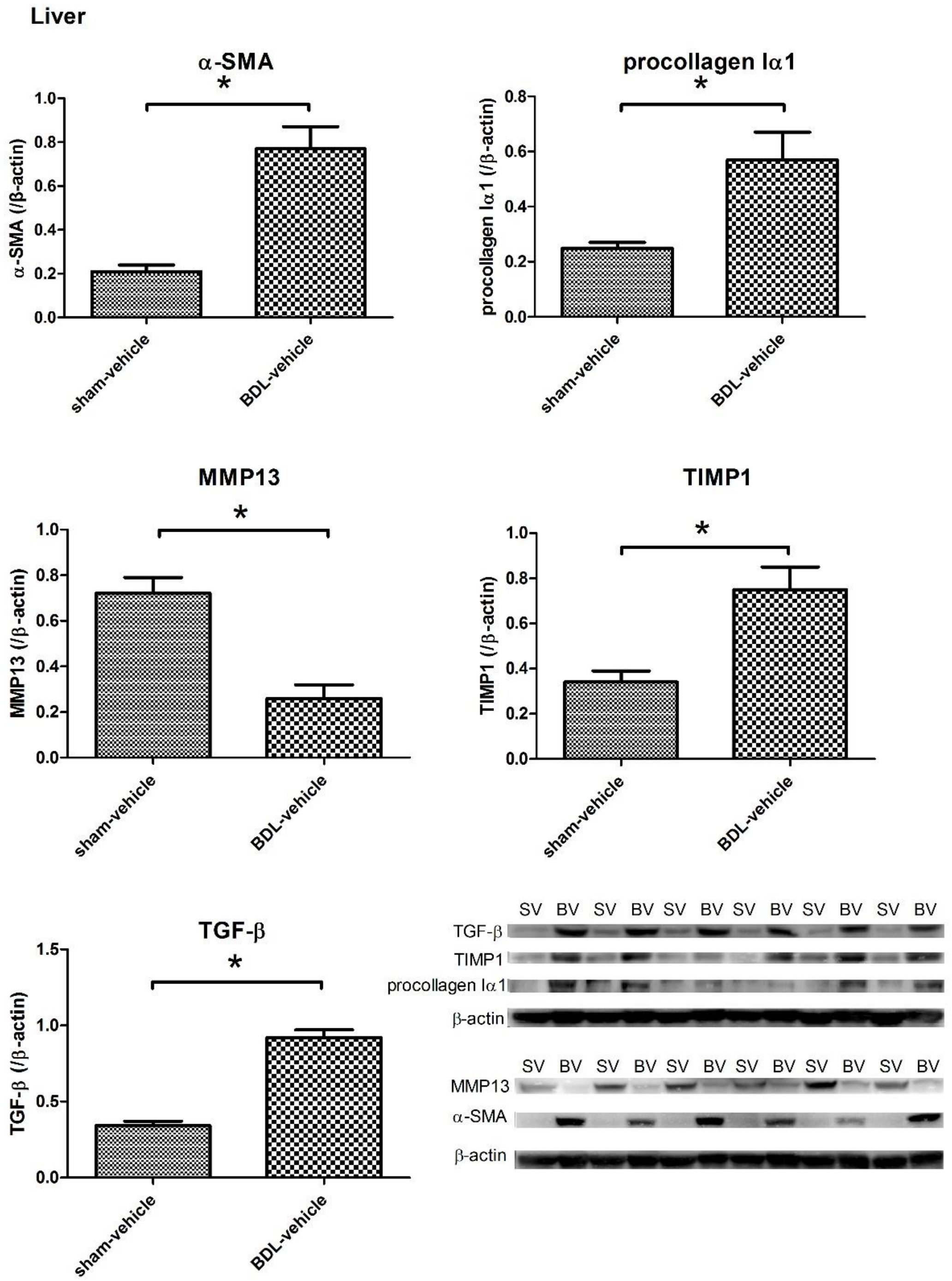

Supplementary figure S3. The fibrogenic protein expressions in the liver. The protein expressions of α–SMA, procollagen Iα1 MMP13, TIMP1 and TGF-β were significantly different between Sham-vehicle (SV) and BDL-vehicle (BV) groups (n=6, 6). * $P<0.05$

Supplementary figures S4. Full uncropped Western blots of liver (BDL-vehicle and BDL-glycyrrhizin groups)

Liver α-SMA

Primary antibody: Genetex, GTX100034

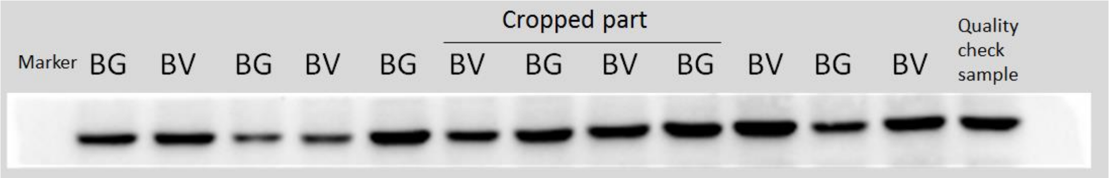

Liver procollagen Iα1

Primary antibody: Abcam, ab34710

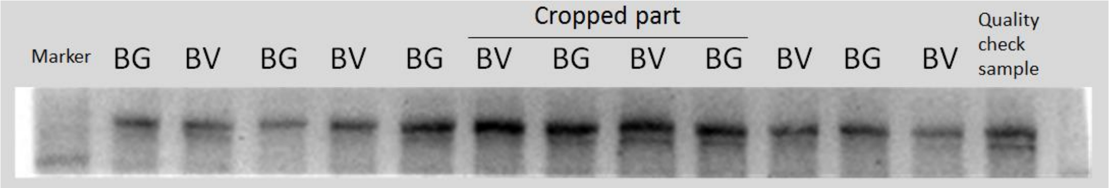

Liver MMP13

Primary antibody: Genetex, GTX100665

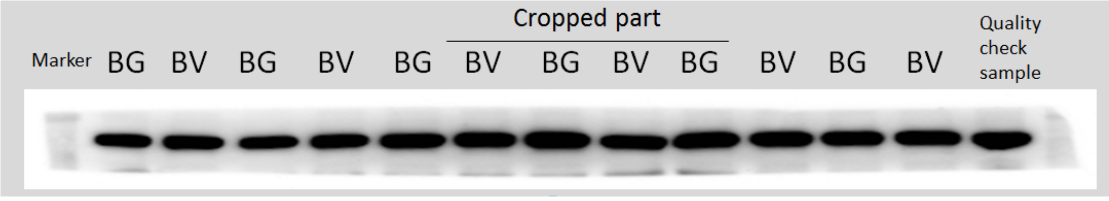

Liver TIMP1

Primary antibody: Genetex, GTX64360

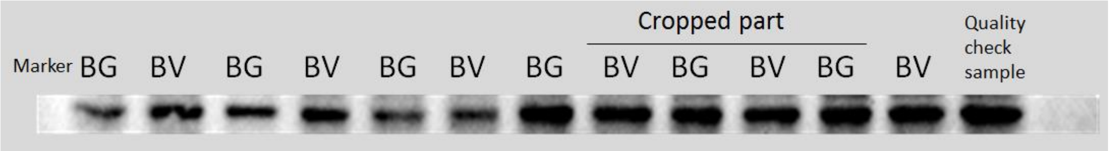

Liver TGF-β

Primary antibody: Genetex, GTX45121

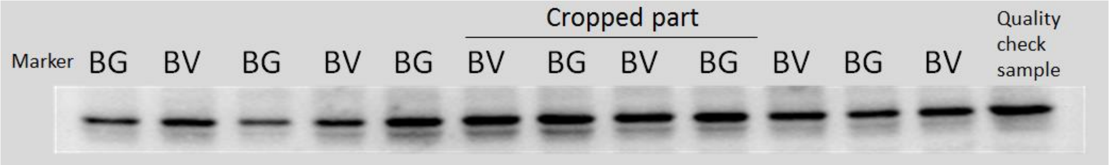

Liver β-actin

Primary antibody: Genetex, GTX629630

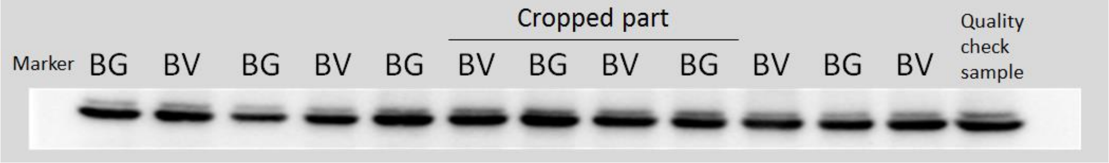

Supplement: Supplementary file 1 [file ijms-22-07662-s001.zip › ijms-1204619-supplementary.pdf]
